# *Zachsia zenkewitschi* (Teredinidae), a Rare and Unusual Seagrass Boring Bivalve Revisited and Redescribed

**DOI:** 10.1371/journal.pone.0155269

**Published:** 2016-05-12

**Authors:** J. R. Shipway, R. O’Connor, D. Stein, S. M. Cragg, T. Korshunova, A. Martynov, T. Haga, D. L. Distel

**Affiliations:** 1 Ocean Genome Legacy, Marine Science Center, Northeastern University, Nahant, Massachusetts, United States of America; 2 Tufts Medical Centre, Boston, Massachusetts, United States of America; 3 The Institute of Marine Sciences, School of Biological Sciences, University of Portsmouth, Portsmouth, P04 9LY, United Kingdom; 4 Koltzov Institute of Developmental Biology RAS, 26 Vavilov St, Moscow, 119334, Russia; 5 Zoological Museum, Moscow State University, Bolshaya Nikitskaya St. 6, Moscow, 125009, Russia; 6 Toyohashi Museum of Natural History, 1–238 Ôana, Ôiwa-chô, Toyohashi, Aichi, 441–3147, Japan; Australian Museum, AUSTRALIA

## Abstract

The sea-grass borer *Zachsia zenkewitschi* belongs to a group of economically and ecologically important bivalves, commonly referred to as shipworms. The sole recognized representative of the genus *Zachsia*, this species displays an unusual life history and reproductive strategy that is now understood to include: environmental sex determination of free swimming larvae, extreme sexual and size dimorphism between males and females, internal fertilization, maintenance of often large harems of male dwarfs within a specialized cavity of the female mantle, and complex maternal care of larvae in specialized brood pouches within the gill. It is also the only shipworm species known to burrow in sea grass rhizomes rather than terrestrial wood. Although *Z*. *zenkewitschi* is rare and little studied, understanding of its biology and anatomy has evolved substantially, rendering some aspects of its original description inaccurate. Moreover, no existing type specimens are known for this species. In light of these facts, we designate a neotype from among specimens recently collected at the type location, and undertake a re-description of this species, accounting for recent reinterpretation of its life history and functional anatomy.

## Introduction

*Zachsia zenkewitschi* is a member of the bivalve family Teredinidae. Commonly referred to as shipworms, Teredinidae is comprised largely of wood-boring species with wide-ranging economic and ecological impacts in coastal marine systems [1]. The teredinid’s ability to bore into and digest wood is estimated to cause billions of dollars in damage per year to coastal constructions, such as piers, jetties, wharfs, fishing and aquaculture equipment [1, 2]. Although often considered pest species, teredinids play fundamental roles in carbon cycling in marine and brackish environments by degrading lignocellulose in floating or deposited wood [3] and in the wood of living mangroves [4].

In most respects, *Z*. *zenkewitschi* exhibits morphological characteristics typical of the family Teredinidae. Due to their unique anatomical characteristics and specialized wood-boring lifestyle, the Teredinidae are among the most highly modified Bivalvia [3, 5]. Unlike more typical bivalves, the double-hinged shell of shipworms covers only the extreme anterior end of the animal, leaving most of the visceral mass exposed. Rather than offering protection to the animal, the shipworm shell, which is covered in tiny denticulated ridges, functions as a drilling tool used to cut into wood. The body is long, vermiform and protected by a calcareous tube, which encloses the animal, except at the anterior and posterior ends where the shell valves and siphons protrude. The siphons are the only parts of the animal that are visible from the surface of the wood. A pair of calcareous, paddle-shaped structures known as the pallets, flank the siphons. These structures, which serve to plug the entrance to the burrow when the animal is disturbed, are unique to, and characteristic of, this family [1, 3].

**Fig 1:**
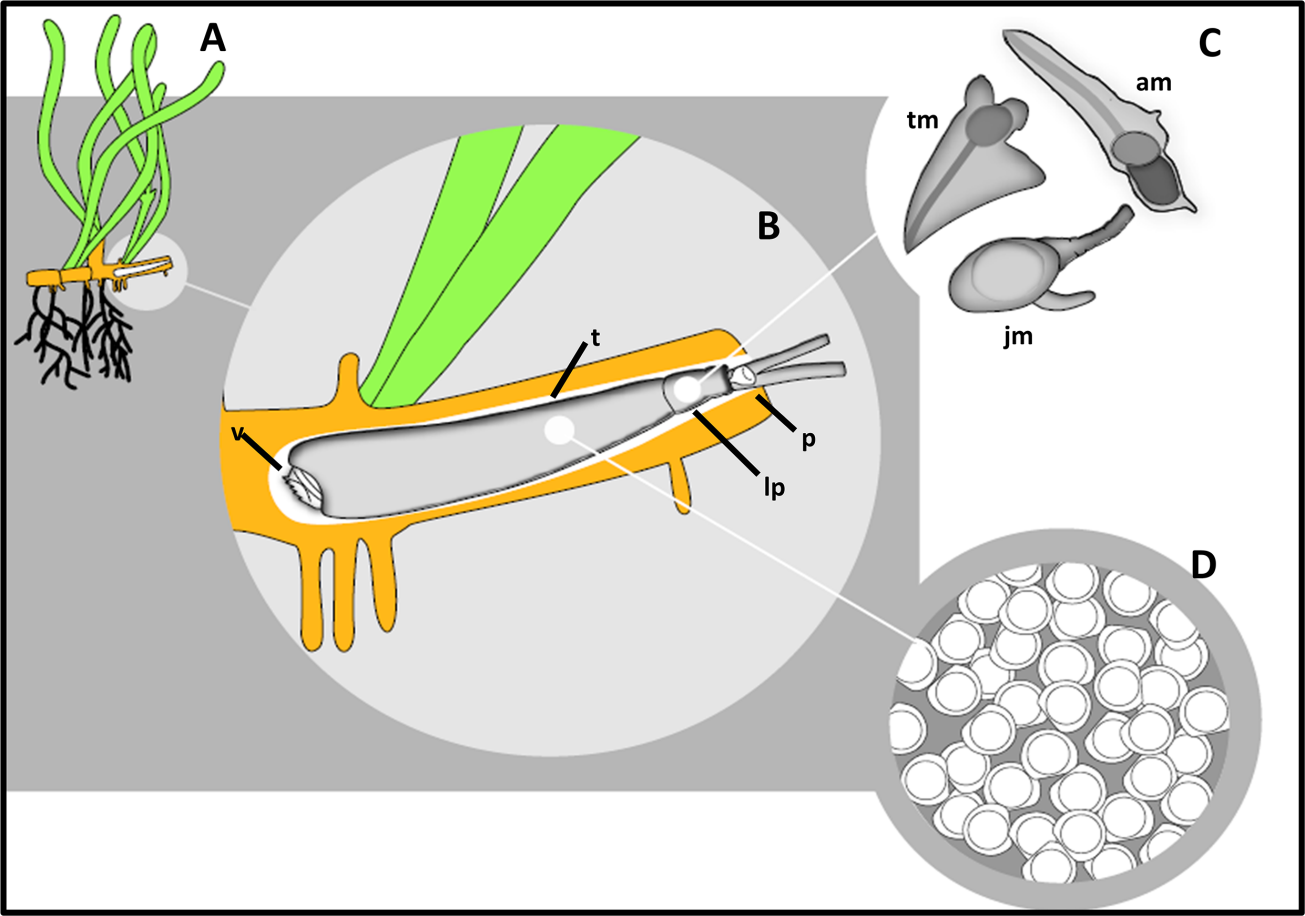
Diagram showing the life stages of the rhizome-boring bivalve *Zachsia zenkewitschi*. A). Location of a burrow of *Z*. *zenkewitschi* in sea grass. B). Detail of a mature female *Z*. *zenkewitschi* in burrow, including: the denticulated shell valve (v), specially modified for burrowing into sea grass rhizomes; the calcareous tube (t) lining the burrow; and the calcareous pallets (p), paddle like structures unique to the Teredinidae, which function to plug the entrance to the burrow. C). A magnified diagram showing males across various developmental stages, from recently metamorphosed juvenile (jm), trefoil stage male (tm) and a fully mature adult male (am), within the lateral pouch of the mature female (lp). D). Brooded larvae, retained in pouches on the maternal gill at the straight-hinged veliger stage.

*Zachsia zenkewitschi* differs from other Teredinidae in several key respects. In contrast to other Teredinidae, which are primarily known to burrow in wood of terrestrial origin, *Z*. *zenkewitschi* burrows in the rhizomes of the sea grasses *Zostera* and *Phyllospadix* [1, 6–8] (Fig 1). In addition, *Z*. *zenkewitschi* exhibits a life history strategy that is arguably the most complex and least understood in this family. It is the sole member of Teredinidae known to exhibit male dwarfism, a form of extreme sexual and size dimorphism [9]. Also, unlike most Teredinidae, which reproduce by broadcast spawning, *Z*. *zenkewitschi* exhibits internal fertilization and short-term larval brooding [9]. In this respect, it resembles members of the genera *Teredo* and *Lyrodus* where larvae develop in specialized brood pouches until release at either the straight-hinged veliger or pediveliger stage [10]. While extreme sexual dimorphism and larval brooding are uncommon amongst the Bivalvia [9], a strategy combining both male dwarfism and complex parental care during larval brooding has, to our knowledge, been described only in this species.

Currently, *Z*. *zenkewitschi* is the sole recognized representative of the genus [11]. Nonetheless, it has been hypothesized that other *Zachsia* species exist. Indeed, seagrass borers morphologically similar to *Z*. *zenkewitschi* have been reported in Vietnam [12] and Papua New Guinea [13], but have not yet been formally described. Taxonomic characterization of these and other new specimens is hampered by the following facts: no type material was designated in the original species description and no suitable materials were found in existing collections; images in the original description were reproduced at low resolution, making meaningful taxonomic comparisons difficult; and key features of the anatomy of shells and pallets are not described. For these reasons, we undertake a re-description and re-evaluation of this species, in which we: collect numerous specimens from multiple geographic locations, including the type location; designate a neotype specimen; evaluate existing museum specimens; and deposit reference DNA and DNA sequences from the neotype to public repositories. Finally, we review advances in understanding of the biology of this species, updating where appropriate the identification of anatomical features and life stages.

## Methodology

### Collection and Imaging

**Fig 2:**
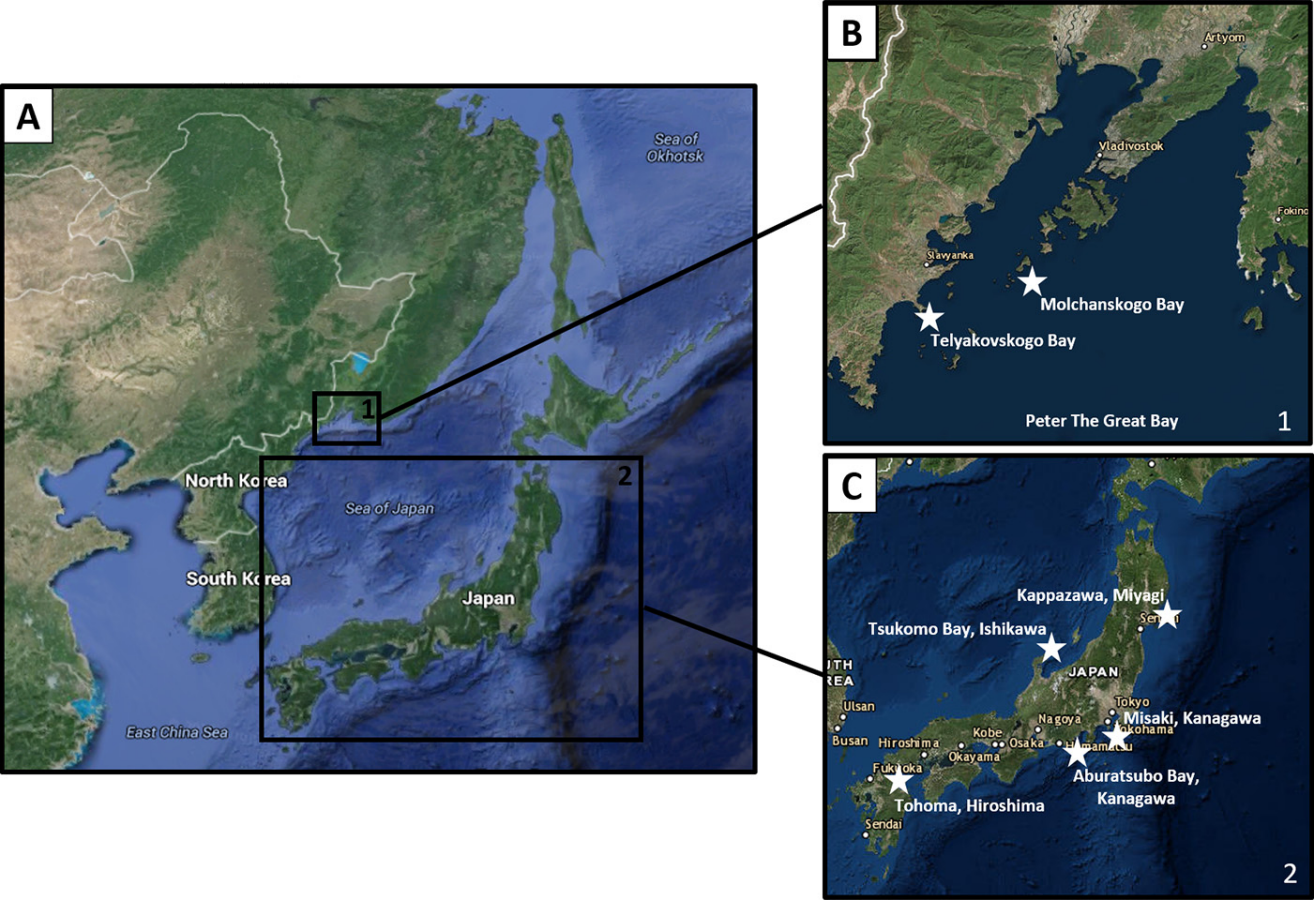
Collection Locations. A). Locations of collection of *Zachsia zenkewitschi* specimens. B). Detail from box 1 in A showing collection location of neotype and topotype specimens in Peter the Great Bay. C). Detail from box 2 in A showing collection locations in Japanese waters. Map generated using Trackline Geophysical Data viewer https://maps.ngdc.noaa.gov/viewers/geophysics/ National Centers for Environmental Information, NOAA. 2016.

Specimens of *Zachsia zenkewitschi* were collected from various locations around the Sea of Japan and the original distribution range from Peter the Great Bay (type locality) [6] (Fig 2). No permits were required for collections at either Japanese or Russian locations. Specimens obtained by SCUBA diving at Shizugawa Bay, Minami-sanriku, Miyagi, were collected under the permission of the Shizugawa Branch, Miyagi Prefectural Fisherman's Union and Shizugawa Nature Center. Permission for the scientific work in the Far East Marine Biosphere Reserve was issued by its director S.M. Dolganov. Samples were fixed in either 4% paraformaldehyde or preserved in absolute ethanol for morphological or molecular analysis, respectively. Specimens were then deposited in the Harvard Museum of Comparative Zoology (Massachusetts, USA), with the Ruth Turner collection (accession numbers MCZ 384428 –MCZ 384435). All specimen details are provided in Table 1. Images were taken using a Nikon SMZ-U dissecting microscope and a Nikon Eclipse E800 compound microscope (Tokyo, Japan). Larval shell dimensions were measured using ImageJ (n = 200) [14].

**Table 1:** *Zachsia zenkewitschi* specimen details. Including details on sample collection (location, substrate, collector and collection date), the accession numbers for samples deposited at the Harvard Museum of Comparative Zoology including type specimens, the GenBank accession numbers for nuclear small (18S) and large (28S) subunit rRNA sequences and the accession numbers for reference DNA samples deposited to the Ocean Genome Legacy biorepository.

Specimen	Location	Substrate	Collector	Date	MCZ ACCN	GenBank ACCN (18S/28S)	OGL ACCN
358230	Vostok Bay, Russia	Unknown	Y. Yakovlev	Aug-79	358230	–	–
HPC 2970	Minami-sanriku Town & Ishinomaki City	*Zostera marina*	T. Haga	27-Nov-05	384428	KU578012/ KU578016	E24247
HPC 1587	Misaki Marine Biological Station, Kanagawa, Japan	*Zostera marina*	T. Haga	24-Aug-07	384429	–	–
HPC 2966	Tohama, Hiroshima, Japan	*Zostera japonica*	Y. Hamamura	15-May-07	384430	KU578011/ KU578015	E24248
Topotype I	Peter the Great Bay, Bolshoi Pelis Island, Molchanskogo Bay, Russia	*Zostera marina*	T. Korshunova, A. Martynov	09-Sep-14	384431	KU578009/ KU578013	E24249
Neotype, Topotype II	Telyakovskogo Bay, Russia	*Phyllospodix iwatensis*	T. Korshunova, A. Martynov	12-Sep-14	384432	KU578010/KU578014	E24250
HPC 2969	Kappazawa, Miyagi, Japan	*Zostera caulescens*	A. Dazai, K. Tanaka, T. Haga	26-Nov-05	384433	–	–
HPC 3030	Aburatsubo Bay, Kanagawa, Japan	*Zostera* sp.	Y. Hayase	05-Nov-01	384434	–	–
HPC 2968	Tsukumo Bay, Ishikawa, Japan	*Zostera* sp.	H. Namikawa	13-Oct-01	384435	–	–

### DNA Extraction and Amplification

DNA was extracted from siphonal tissue and associated musculature from four specimens of *Z*. *zenkewitschi* (specimen details found in Table 1). Total genomic DNA was extracted using the DNeasy Blood & Tissue kit (Qiagen). Approximate concentration, and yield of DNA were determined by UV spectrophotometry. Genomic DNA was cryo-preserved at – 80°C and archived at the Ocean Genome Legacy Center of New England Biolabs, Northeastern University, Nahant, MA, USA (accession numbers in Table 1).

The small and large subunit nuclear rRNA genes were amplified from the resultant DNA preparations by polymerase chain reaction (PCR). Amplification reactions were prepared using 12.5μL of high fidelity polymerase solution (OneTaq®, New England Biolabs, Ipswich, Massachusetts), 0.5 μL of each primer (10 mM), 1–2 μL DNA template (10–20 ng/μL), brought to a total volume of 25 μL with purified water. Fragments of the large (28S) and small (18S) subunit nuclear rRNA genes were amplified using the primer pairs 18S EukF (Forward 5' WAY-CTG-GTT-GAT-CCT-GCC-AGT 3'), 18S EukR (Reverse 5' TGA-TCC-TTC-YGC-AGG-TTC-ACC-TAC 3') [15], 28S-NLF184-21 (Forward 5' ACC-CGC-TGA-AYT-TAA-GCA-TAT) and 28S-1600R (Reverse 5’ AGC-GCC-ATC-CAT-TTT-CAG-G) [16], resulting in amplicons of approximately 1686 and 1416 base pairs respectively.

The thermal regime for the PCR amplifications proceeded as follows: an initial denaturation step of 94°C for three minutes, followed by 35 cycles with a denaturation step of 94°C for 20 s, an annealing step of 64°C for 40 s for the 18S and 63°C for 30 s for 28S, an extension step of 68°C for 60 s and a final extension of 68°C for five minutes. All reactions were performed on a PTC-200 Thermal Cycler (MJ Research, Quebec, Canada).

For each template, three separate amplicons, all produced under identical conditions, were pooled, cleaned and concentrated using the Zymo Clean & Concentrator Kit (Irvine, CA). Resulting products were sequenced bidirectionally on a 3730 XL DNA Analyzer (Life Technologies, Grand Island, NY) using the Big Dye Terminator 3.1 Cycle Sequencing Kit (Life Technologies, Grand Island, NY) at New England Biolabs (Ipswich, Massachusetts). Primary sequence data was submitted to GenBank (NCBI) under accession numbers reported in Table 1.

## Results

### i) Taxonomy

**Fig 3:**
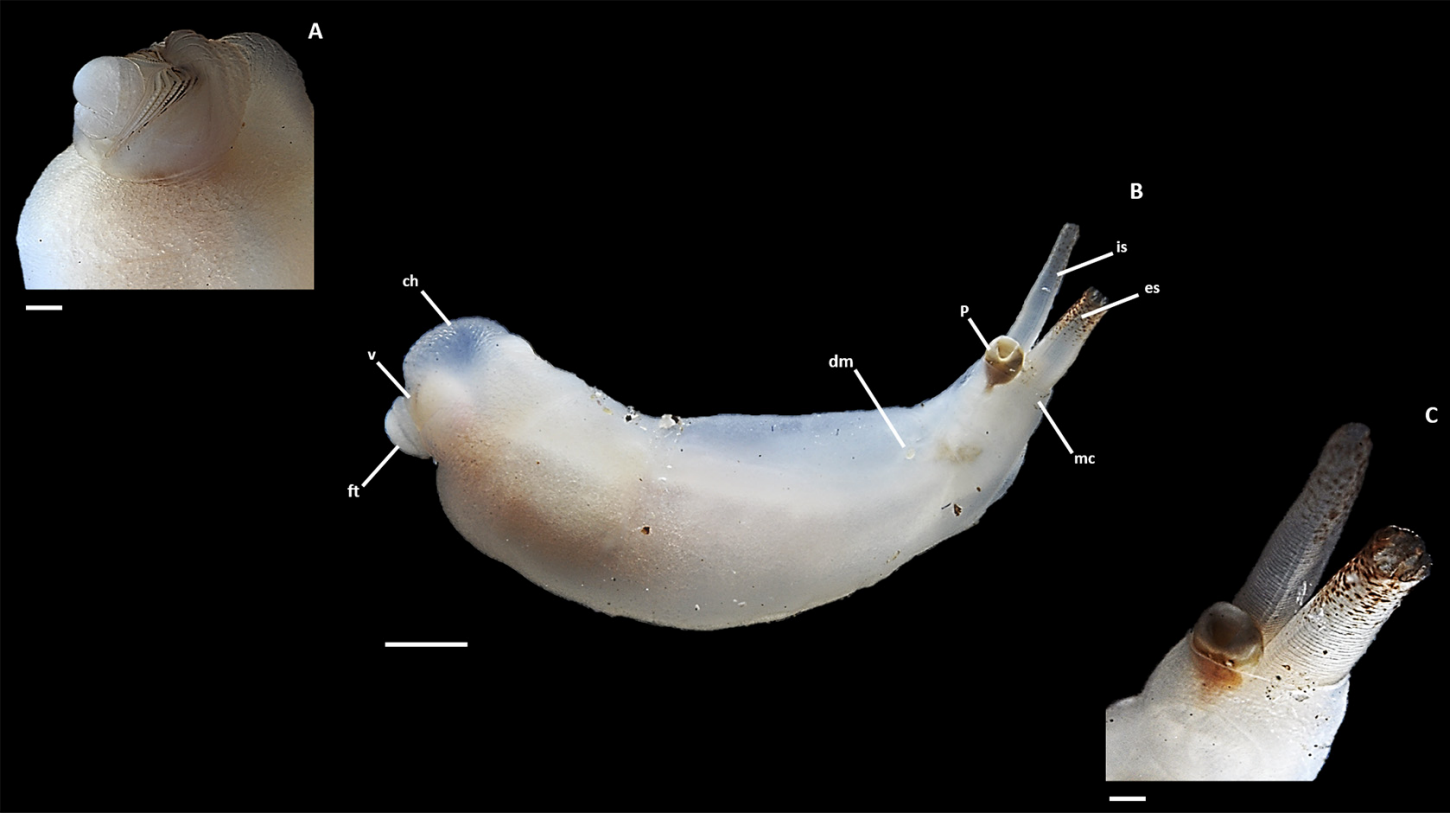
Specimen of *Zachsia zenkewitschi* (Neotype) collected from Telyakovskogo Bay, Russia. A). Detail of anterior region. B) Complete specimen removed from burrow. C) Detail of posterior region. (ch), cephalic hood; (dm), dwarf male; (ft), foot; (mc), mantle collar; (p), pallet; (es) excurrent siphon (is), incurrent siphon; (v), valve. Scale bars equal to 1 mm (A, C) and 5 mm (B).

Class Bivalvia Linnaeus, 1758; Order Myoida Stoliczka, 1870; Family Teredinidae; Genus Zachsia Bulatoff & Rjabtschikoff, 1933; Type species: *Zachsia zenkewitschi* Bulatoff & Rjabtschikoff, 1933, by original designation (Fig 3).

### ii) Material Examined

#### Neotype MCZ 384432 (Fig 3)

67 mm in total body length and 7 mm in width. Found within the rhizomes of the seagrass *Phyllospodix iwatensis*. Specimens were collected on the 12th of September 2014 from Telyakovskogo Bay, Peter the Great Bay, Primorye along the Sea of Japan, Russia, 42°35'17.09"N 131°13'14.08"E, fixed in 4% paraformaldehyde and stored in 70% ethanol (Table 1).

**Other Specimens:** Multiple specimens from the collections in the Sea of Japan and from the Harvard Museum of Comparative Zoology, Massachusetts, USA were examined. Specimen details are listed in Table 1.

### iii) Diagnosis

#### General Morphology

Shell valves are small and cover approximately half the width of the anterior region (Fig 3A and 3B). The incurrent siphon is approximately double the girth and half the length of the excurrent siphon, both of which are partially tinged with a reddish brown pigmentation (Fig 3B and 3C). The mantle collar envelops the pallets midway up the blade. The gills extend three quarters the length of the animal, reaching to the posterior edge of the posterior adductor muscle at a point near the base of the siphons. Mature female *Zachsia* retain larvae until the straight-hinged veliger stage in brood pouches on the dorsolateral surface of the gill (Fig 3A and 3B). Dwarf males, across various developmental stages are found in a specialized region of the female mantle, formed from a fold of the of the mantle collar (Fig 3). This region is located immediately anterior to the siphon, extending only to the beginning of the gill. The anterior region of *Zachsia* differs markedly from other teredinids with the cephalic hood and foot comparatively enlarged and the shell valves reduced. The translucent cephalic hood, once distended, is approximately double the size of the shell valves (Fig 3A and 3B). The foot is bulbous and extends beyond the aperture of the shell valves (Fig 3A and 3B).

#### Description of calcareous structures

**Fig 4:**
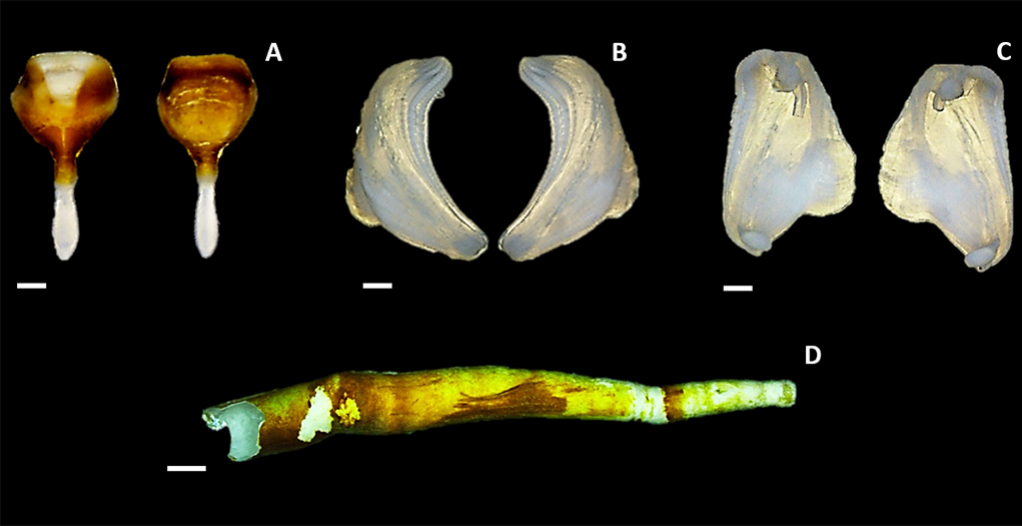
The calcareous structures of *Zachsia zenkewitschi*. A). The outer and inner margins of the calcareous pallet removed from specimen MCZ 384428. B, C). The outer and inner surfaces of paired shell valves removed from specimen MCZ 384428. D). The calcareous burrow of *Zachsia zenkewitschi* (MCZ 384434) removed from the rhizome of the seagrass of *Zostera sp*. Scale bars equal to 1 mm (A—C), 5 mm (D).

Pallets are short and broad. The calcareous portion appears diamond shaped in lateral view (Fig 4A). The outer margin is characterized by a distinct thumbnail depression caused by a deep U-shaped concavity. This gives the distal portion of the pallet a hollow appearance (Fig 4A). The inner margin bears only a slight concavity. A golden-brown periostracum crowns the proximal portion of the pallet, particularly on the outer margin. This periostracum thickens laterally on the inner margin and forms a thin perimeter around the pallet. The stalk is short, straight and rounded at its distal end (Fig 4A).

The valves of *Z*. *zenkewitschi* are narrow with an angular, nearly L-shaped, appearance (Fig 4B and 4C). There is little distinction between the anterior and median lobes. Denticulated ridges gently merge between the two lobes, with the densest concentration located towards the umbonal knob. The valves are small with respect to body size and appear angular due to the very slim anterior slope, the highly reduced posterior slope and the small auricle (Fig 4B and 4C). Only one type of denticle is present. These denticles are large with rounded apices, projecting at an angle nearly perpendicular to the valve surface anteriorly in the direction of boring. The calcareous burrow (Fig 4D) lines the inner surface of the bored rhizome and is similar to those of other teredinids.

### iv) Habitat & Distribution

All *Z*. *zenkewitschi* samples observed to date have been found in the rhizomes of the seagrasses *Phyllospadix* and *Zostera* with patchy distribution across the Primorsky Region (Russia) and Ishikawa Prefecture (Japan) in the Sea of Japan [8, 17, 18] and on the Pacific coast of Japan [7]. However, live specimens have been recovered from drifting seagrass rhizomes, possibly extending beyond the known distribution range.

### v) Reproductive & Life History Strategy

**Fig 5:**
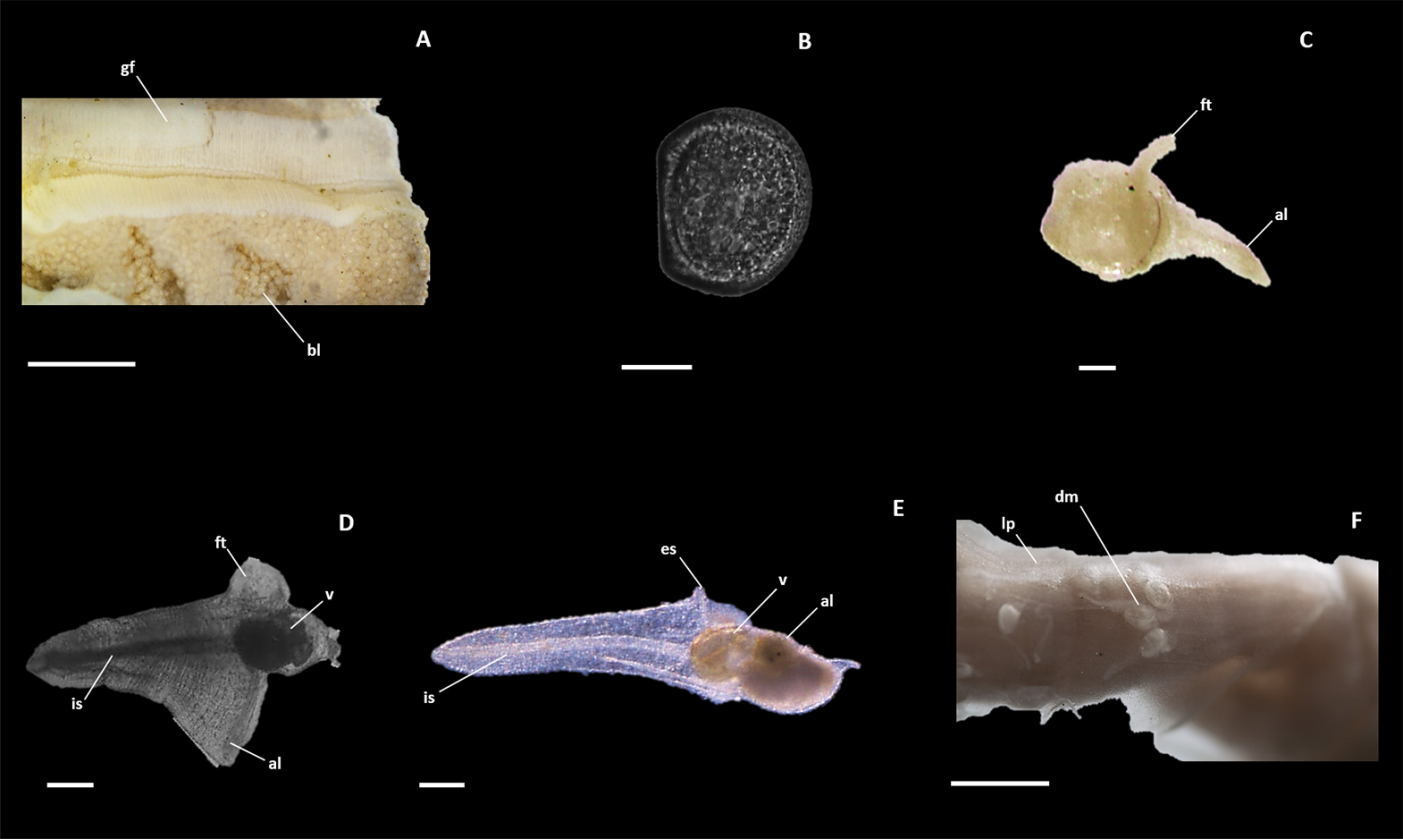
Life stages of *Zachsia zenkewitschi*. A). A region of gill showing modified brood pouches containing D-stage veliger larvae. B). D-staged veliger removed from the parental brood pouch. C). Juvenile male at the early stage of metamorphosis. D). Trefoil stage male mid-way through metamorphosis. E). Fully mature adult male. F). Mature males residing within the lateral mantle pocket of a mature female; (al), anterior lobe; (bl), brooded larvae; (dm), dwarf male; (es), excurrent siphon; (ft), foot; (gf), gill filament;(is), Incurrent siphon; (lp), lateral pouch; (v), valve. Scale bars equal to 500 μm (A,F) and 100 μm (B-E).

Large numbers of D-shaped veliger larvae were found within specialized brood pouches on the gills of a number of specimens (Fig 5A and 5B). Measurement of larval shells (N = 200) revealed similar dimensions with an average shell length of 75 μM (± 10 μM) and a shell width of 65 μM (± 9 μM). Dwarf males were clearly visible in the lateral pouch of the mantle, located immediately anterior to the female siphon (Fig 5C–5E). (Fig 5F). Multiple individuals, across all developmental stages—from pediveliger larvae at the beginning of metamorphosis, to trefoil stage larvae and mature reproductive males—were found to co-occur within the female. Most males were oriented with their incurrent siphon facing towards the female incurrent siphon.

### vi) Molecular Sequence Data

Bidirectional sequences were obtained for both the small (18S) and large (28S) subunit nuclear rRNA genes, from four specimens of *Zachsia zenkewitschi* (MCZ 384428, MCZ 384430, MCZ384431/Topotype I and MCZ 384432/Topotype II). These specimens represent two distinct geographical ranges: (a) the original distribution range (Topotypes I and II) in Peter the Great Bay, including a specimen from the same collection as the Neotype and (b), the Pacific side of Japan (MCZ 384433 and MCZ 384428) (Fig 2). Sequences determined for each locus were identical among all specimens examined. Using the Basic Local Alignment Search Tool (BLAST) to search GenBank (1^st^ December 2015), sequences for the both loci were confirmed to be most similar to those of other members of the family Teredinidae. The phylogenetic position of *Zachsia zenkewitschi* with respect to other teredinid species is part of an ongoing investigation that will be published independently.

## Discussion

The taxonomy, morphology, biogeography, ecology, life history and reproductive strategies of *Z*. *zenkewitschi* are poorly understood, due, at least in part, to the rarity of this species and the consequent scarcity of opportunities for observation. Taxonomic descriptions of *Zachsia* are absent from most extensive reviews of the family Teredinidae, e.g. [1] and [3]. The limited information currently available to describe *Z*. *zenkewitschi* is fragmented throughout a relatively obscure literature. In many cases, including that of the original species description [6], these manuscripts are found in older volumes of German, Japanese or Russian language journals that are difficult to obtain except as low resolution scans of aging printed materials. Moreover, the original species description predates most modern taxonomic treatments for the family Teredinidae and lacks detailed description of the valves and pallets, the latter of which is now among the features considered most important for species identification.

Similarly, the original description predates current interpretations of the biology and life history of the species and as a result misidentifies a key life stage and related anatomical features. In the original description, two locations for brooded larvae were identified, one in brood pouches on the gill and a second in a pouch located at the posterior end of the mantle near the mantle collar. The latter was reported to contain unusual “tailed larvae”. Rather than offspring of the female on which they are found, these individuals are now recognized as mature adult dwarf males and are likely the offspring of other females. Therefore, the sac that contains them, initially incorrectly identified as a brood pouch in the original description, is referred to hereafter as the lateral pouch.

The shortcomings of the original species description are compounded by the apparent lack of existing type specimens for this species. Type specimens were neither designated nor their whereabouts described in the original species description. Turner [1] reported that the holotype specimen was deposited to the Zoological Museum of Moscow State University, however, our inquiries to this collection, and numerous others to which the original authors may have had access, found no record of this accession. All specimens listed for *Z*. *zenkewitschi* by the Global Biodiversity Information Facility (GBIF) as of the time of this writing (December 2015), are attributed to the Museum of Comparative Zoology (Harvard University, Cambridge, MA, USA). Inspection of these specimens revealed them to be in a deteriorated condition, poorly suited for morphological or molecular analysis. Thus, the collection of new specimens of *Z*. *zenkewitschi* from the type location and other locations by the authors provided an opportunity to designate a neotype specimen, and to update the description of this rarely observed species based on fresh materials, taking into account recent literature.

The taxonomic approach undertaken herein, includes the first DNA sequence data reported for *Zachsia zenkewitschi*. Importantly, we provide sequence data from specimens collected in Peter the Great Bay—the originally described distribution range—including the location of the Neotype specimen. These sequences were compared with those of *Z*. *zenkewitschi* specimens collected from the Pacific coast of Japan (HPC 2969 and HPC 2970). No detectable differences were observed in the sequences of the examined biomarkers (small (18S) and large (28S) nuclear rRNA genes).

Many aspects of the anatomy and life history of *Z*. *zenkewitschi* appear to be influenced by its unusual habitat preference. Unlike the woody substrates inhabited by most Teredinidae, the seagrass rhizomes are characteristically soft and are of very narrow diameter (typically no more than a few millimeters). This likely poses unique problems for this species. For example, the burrowing action utilized by most Teredinidae both widens and lengthens the burrow [4, 19]. However, because the diameter of sea grass rhizomes is so restricted, a strong ability to widen the burrow may not be necessary, or even desirable. Continual widening of the burrow would quickly exceed the diameter of the rhizome, breaking its delicate structure and preventing further extension of the burrow. This would not only cut off the food supply, but would also subject the animal to increased threats related to exposure, including predation and desiccation.

Modifications observed in the valve structure of *Z*. *zenkewitschi* may be adaptive in this respect. The shell valve apparatus in most Teredinidae is a highly modified structure, serving as an effective tool for boring through wood. Most teredinids have two distinct sets of tiny teeth-like denticles, one on the anterior slope which functions to lengthen the burrow at the excavation face, and a second on the median slope which functions to widen the burrow. Large muscle insertions for the posterior adductor muscle on the auricle, and for the foot on the lever-like apophysis, facilitate a powerful scissor-like motion of the valves that drives the denticles across and into the surface of the wood, the rear-facing orientation of the denticles helping to drive the animal toward the excavation face during each cutting stroke [19].

The shells of *Z*. *zenkewitschi* differ markedly from those of other teredinids, with only one denticle type present. These teeth project perpendicular to the surface of the anterior slope, rather than tangentially as in most Teredinidae, and face toward, rather than opposite to, the direction of boring. The reduced size and absence of denticles on the median slope suggest a reduced capacity for widening the burrow. This likely allows the burrow width to remain relatively constant as the burrow extends, thereby reducing the risk of exceeding the diameter of the rhizome. In addition, the greatly reduced auricle and posterior adductor attachment, in combination with the forward facing orientation of the shell teeth, suggests a burrowing stroke quite different from that of other Teredinidae [7], possibly reflecting the differing mechanical properties of the rhizome material as compared to wood.

The unique challenges of life in a narrow sea grass rhizome may also be reflected in the life history of *Z*. *zenkewitschi*. In the case of these animals, the narrow burrow not only restricts available space for growth and reproduction, but also restricts the availability of food. It has been proposed that, in cases where both food and space are limited and the female is sessile, extreme sexual dimorphism (male dwarfism) may have an adaptive advantage. The smaller males may require less food and space, thereby reducing competition for these resources between the sexes [9, 20].

Like other teredinids, female *Zachsia* likely continue to grow throughout their life cycle, limited only by the availability of substrate into which they burrow. Conversely, males exhibit arrested development shortly after metamorphosis and retain certain larval characteristics, thus leading to pronounced differences in size and morphology between the sexes. This size discrepancy allows individual females to support multiple dwarf males. Up to 105 males have been reported on a single female [8, 10]. As previously observed, males of multiple developmental stages ranging from recently settled pediveligers to fully developed and sexually mature dwarves, were observed within individual females [9]. The co-occurrence of developmental stages indicates continued recruitment of males to the harem throughout the female’s lifespan.

The male dwarves are sheltered in a specialized structure formed from a fold of the mantle collar [7] and located anteriorly to the siphons. Like the dorsal lappets found in species of *Neoteredo* [21], this ‘lateral pouch’ is unique to *Zachsia* and may be informative as a taxonomic character. The lateral pouch has no direct access to the female epibranchial cavity, thus males cannot provide sperm directly to the female. Instead, males produce sperm balls, which are released into the water column [9, 22]. The close proximity of the lateral pouch to the incurrent siphon of the female ensures that sperm is swiftly taken up into the mantle cavity for internal fertilization [8, 10, 22]. Despite their brief external existence, the male gametes share characteristics with spermatozoa of internally fertilizing species [23]. It is likely that packaging the sperm into sperm balls helps to reduce dispersal in the water column and so increases the likelihood of delivery to the female on which the male resides.

The presence of multiple mature males in the lateral pouches likely increases both sperm provision to the female and fertilization success. While it is not known whether the male and female synchronize gamete spawning, all larvae from a given individual in this study were found to display uniform size and developmental state, consistent with observations of other short-term brooding teredinids [3]. As larval growth post-fertilization follows a typical developmental time-line [24], this synchronized larval development suggests fertilization from a single event. This could be achieved either by synchronous periodic release of gametes by male and female, or by periodic release of female gametes with continuous sperm production by mature males. Once released from the parent, veligers must undergo a significant planktotrophic phase to complete larval development. Among other short-term brooders, this stage typically lasts between two to three weeks [1, 24, 25]. This strategy results in a shorter planktonic lifespan compared to broadcast spawners, potentially subjecting larvae to lower risk of mortality during this vulnerable stage [26] while still allowing adequate dispersal away from the parent population [25].

It has been proposed, that sex in *Z*. *zenkewitschi* is environmentally determined, with larvae that settle on rhizomes becoming female and larvae that settle on females becoming male [9]. It is noteworthy that all other species of Teredinidae are thought to be protandrous hermaphrodites. Thus a more parsimonious hypothesis might be that larvae settling on rhizomes may experience a brief or truncated male stage, while larvae that settle on females may experience arrested development in the male stage. The large number of males observed on each female, and their presence in various developmental stages, suggests that free-swimming larvae are attracted to the female throughout the lifespan of the mature female, possibly via chemical attractants. Larvae might also be attracted to rhizomes via chemical attractants. Indeed, it has been shown that shipworm larvae can find wooden settling substrates through the detection of waterborne chemical cues [27].

It remains to be determined how this potential competition between chemical cues influences the biogeographic distribution of this species. The large number of males found on each female suggests that planktonic larvae carried within range of a mature female are strongly recruited to metamorphose within her mantle cavity to become males, thus concentrating a high abundance of individuals within a single rhizome. Pioneering larvae that range beyond the catchment area of a mature female, become female but risk dispersing too far from the larval pool and so may never recruit larvae to become mating dwarf males. Moreover, larvae that are carried beyond the range of a sea grass habitat will fail to complete metamorphosis entirely. Thus, this strategy may place significant constraints on dispersal and may account for the narrow distribution and rarity of this enigmatic species.

In summary, the presence of few female borers per rhizome likely reduces intraspecific competition and maximizes utilization of this food and habitat resource. Thus, a single dominant female may grow large, increasing fecundity, larval cohort size and reproductive output. Furthermore, larger females may also support a greater harem of dwarf males, ensuring adequate sperm provision with little competition from males for limited nutrient and spatial resources within the rhizome. Commensalism between females and their dwarf males ensures continual reproduction should fragmentation and rafting of the rhizome occur, facilitating this as an additional means of dispersal [7]. Internal fertilization increases the likelihood of sperm-egg encounters and therefore fertilization success; the subsequent retention and brooding of larvae to the straight-hinged veliger stage protects offspring during the most vulnerable period of the life cycle. Larvae are then released at a developmental stage closer to competency, which enables a shorter planktonic lifespan subject to less mortality; this planktonic stage allows larvae to disperse away from parent populations and ensures a higher likelihood of locating a suitable substrate/mate.
